# Multivariate Analysis of Microbiological and Incubation Parameters in Hatching Eggs Sanitized with or Without Essential Oils

**DOI:** 10.3390/vetsci12070600

**Published:** 2025-06-20

**Authors:** Gabriel da Silva Oliveira, Concepta McManus, Vinícius Machado dos Santos

**Affiliations:** 1 Faculty of Agronomy and Veterinary Medicine, University of Brasília, Brasília 70910-900, Brazil; 2 Center for Nuclear Energy in Agriculture (CENA), University of São Paulo, São Paulo 13416-000, Brazil; 3 Laboratory of Poultry Science, Federal Institute of Brasília—Campus Planaltina, Brasília 73380-900, Brazil

**Keywords:** eggshell contamination, egg sanitization, hatchability, poultry production, sanitizers, yolk sac contamination

## Abstract

Aspects related to the contamination of hatching eggs, sanitary management during pre-incubation, and the performance of the incubation process can compromise productive efficiency in poultry farming. When these factors negatively influence poultry farming, they can destabilize the generation and distribution of financial resources throughout the production chain, as well as limit public access to poultry-derived proteins. Understanding how these aspects are interrelated is essential for making decisions that benefit poultry health and productivity. Therefore, we conducted a multivariate analysis of microbiological and incubation parameters to evaluate whether bacterial contamination of the eggshell and yolk sac negatively affects HI and to compare the effectiveness of different sanitization protocols in reducing bacterial contamination in these regions. To achieve this, we utilized the raw data from our previous research on the sanitization of hatching eggs and conducted a detailed statistical analysis to evaluate the relationships between the studied variables. The correlation analysis revealed that eggshell mesophilic bacterial contamination (EGM) was strongly associated with yolk sac mesophilic bacterial contamination (YSM) (r = 0.76) and yolk sac contamination by Enterobacteriaceae (YSE) (r = 0.73). The principal component analysis indicated a negative association between HI performance and eggshell and yolk sac contamination. Results indicated beneficial associations between the reduction of contamination in hatching eggs and increased hatchability rates when using essential oils. The bacterial load of hatching eggs contributes to reduced productivity, reaffirming the need for proper egg sanitization, especially using essential oils.

## Simple Summary

In the poultry chain, the beneficial interaction between health and productivity must be constant. These elements need to work together to ensure efficient and safe results. For example, the implementation of hatching egg sanitization practices has proven to be an effective strategy for achieving good productivity rates and obtaining healthy chicks. It is essential to understand how these factors correlate to ensure both bird health and maximum productivity. Therefore, we conducted a multivariate analysis of microbiological and incubation parameters to evaluate whether bacterial contamination of the eggshell and yolk sac negatively affects hatchability (HI) and to compare the effectiveness of different sanitization protocols in reducing bacterial contamination in these regions.

## 1. Introduction

Egg contamination is of interest due to its occurrence in non-sterile environments [1], exposure to unsanitary conditions after laying [2], and, most importantly, the effects of this contamination on poultry health. Egg contamination has been found to be associated with yolk sac infection [3]. This contamination has been previously described through two routes: vertical, which occurs during the formation of the egg, meaning bacteria are already present in the egg’s content when it is formed; and horizontal, when the already-formed egg is exposed to bacteria that penetrate the shell and migrate into the contents [3]. Each route of contamination requires a strategic control plan aligned with poultry health.

When it comes to sanitizing hatching eggshells, horizontal bacterial contamination is more relevant than vertical contamination, both from a productive and economic standpoint, as it is the only type that can be directly minimized through the topical application of sanitizer on the eggshell [4]. Reducing surface contamination is critical for four main reasons: (1) reducing the presence of pathogenic microbiota; (2) minimizing complications arising from this microbiota, including infections and embryonic mortality; (3) preventing cross-microbial transmission in the poultry environment; and (4) ensuring that poultry professionals are exposed to minimal microbiological risks in the workplace.

Proper sanitization is a viable option to reduce contamination of eggshells [5]. This process involves the application of synthetic or natural chemical compounds to surfaces, whether they are inanimate or not, in effective doses, following the manufacturer’s recommendations or based on scientific evidence, and according to the specific needs of each stage of poultry production. This procedure is followed by the conservation and monitoring of the sanitized surface. The frequency of sanitization may vary from minutes to months, depending on the production stage. For instance, after egg collection, the sanitation of hatching eggs typically occurs at intervals of minutes, with the application being carried out based on the frequency of collection, the source batch, and the amount that the sanitization environment can accommodate.

The effectiveness of hatching egg sanitization in reducing contamination can vary significantly. In their review, Oliveira et al. [6] compared formaldehyde, the primary compound used for egg sanitization, with essential oils. They demonstrated that both substances exhibit significant antibacterial activity in reducing eggshell bacteria. This is due to the specific antibacterial mechanisms of each. As reviewed by Ricke et al. [7], formaldehyde targets the spore cores and cell walls of bacteria. On the other hand, Li et al. [8] reported that essential oils inhibit bacteria by interacting with the cell membrane, impairing its function and physiological activity through direct interaction with the hydrocarbon chains of the phospholipid tails. However, the antibacterial effects of formaldehyde and essential oils in reducing eggshell contamination are not always similar, and one may be more effective than the other, due to the influence of factors such as the intrinsic characteristics of each (see review by Oliveira et al. [6]).

Sanitizers for hatching eggs can be validated in both laboratory and industrial settings not only based on microbial levels on the eggs, but also on incubation outcomes, or a combination of both [9,10,11,12,13]. The incubation results encompass various parameters, such as egg weight loss, chick characteristics, mortality rates, and, most importantly, hatchability (HI) [14,15]. The latter parameter serves as the primary indicator for assessing the efficiency of the incubation process. In parallel, the microbiological parameters include the microbial count present on the eggshell and in the embryos. Previous studies have indicated that various pre-incubation and incubation factors can influence HI rates [16,17]. However, these studies have not delved into the specific aspects of egg sanitization that truly influence these variations.

This study aims to fill this gap by applying multivariate analysis of microbiological and incubation parameters, exploring the relationship between contamination of the eggshell and yolk sac (mesophilic bacteria and Enterobacteriaceae) with HI in hatching eggs sanitized or not with essential oils. Multivariate analysis is a methodology that allows the simultaneous evaluation of multiple factors, providing a more precise understanding of the relationships between bacterial contamination, sanitization, and HI.

## 2. Materials and Methods

In this study, we used the data matrix generated in our previous research on hatching egg sanitization [18], with approval from the Ethics Committee on Animal Use at the Federal Institute of Brasília (IFB Document Number 6739150424). Briefly, the research involved analyzing the application of different essential oils and control treatments on hatching eggs after collection, namely: (1) Non-sanitized eggs (NE); (2) Spraying with grain alcohol (GA) at a concentration of 93.8%; (3) Fumigation with formaldehyde (FA) at a concentration of 5 g/m^3^; (4) Spraying with *Citrus aurantifolia* essential oil (CAEO) at a concentration of 9.38 mg/mL; (5) Spraying with *Ocimum basilicum* essential oil (OBEO) at a concentration of 4.69 mg/mL; and (6) Spraying with *Allium sativum* essential oil (ASEO) at a concentration of 1.17 mg/mL.

This study used hatching eggs with an average egg weight per replicate ranging from 56 to 60 g from a 57-week-old broiler breeder from the Pescoço Pelado Vermelho (PSÇ) lineage. Except for the control treatment, all eggs were sanitized simultaneously 20 min after collection, then naturally air-dried at room temperature for approximately 30 min. Subsequently, they were stored for 24 h at a temperature between 19 and 21 °C and relative humidity between 50% and 60%. Liquid sanitizers were applied using hand sprayers, whereas the gaseous sanitizing agent was used in a closed environment according to the farm’s standard protocols. For liquid sanitizers, a dose of approximately 2.5–3 mL per egg was applied. After the storage period, eggs were incubated in single-stage setters (Chocmaster, Curitiba, Paraná, Brazil) at an average temperature of 37.7 °C and relative humidity of 60%, with hourly turning throughout the incubation period. During hatching, eggs were maintained at 36.6 °C with an average relative humidity of 65% without turning.

Throughout the experiment, various parameters were performed, including hatchability of fertile eggs (HI, %), which represents the relationship between the number of hatched chicks and the number of fertile eggs; number of contaminated eggs (CE,%) throughout the incubation process; early mortality (EM, %); intermediate mortality (IM, %); late mortality (LM,%); egg weight (EW, g) after storage; egg weight loss (EWL, %) calculated as the relationship between egg weight on day 0 and day 18 of incubation; chick weight (CW, g) after hatching; yolk sac bacterial count (log_10_ CFU/mL) on the 18th day of embryonic development; and eggshell bacterial count (log_10_ CFU/mL) after sanitization.

The raw data from the previous study was used to investigate the relationships between microbiological and incubation parameters, particularly to explore, for the first time in the field of hatching egg sanitization, the correlation between egg contamination and HI, comparing the effectiveness of different sanitization protocols through various statistical correlation analyses. The application of this methodological approach in this area of research aims to provide quantitative evidence to either support or refute previously established assumptions regarding this relationship. Through these analyses, this study seeks to measure the strength and direction of this correlation objectively. The results obtained will enable a more precise understanding of the actual impact of contamination on HI, contributing to developing more effective strategies to mitigate this issue. Additionally, this will enhance the comprehension of the effectiveness of sanitization protocols in reducing contamination.

For this study, the analysis was conducted based on six treatments and four repetitions (each repetition comprising 60 eggs; totaling 1440 eggs) for the following parameters: hatchability of fertile eggs (HI), number of contaminated eggs (CE), early mortality (EM), intermediate mortality (IM), late mortality (LM), egg weight (EW), egg weight loss (EWL), and chick weight (CW). Additionally, the parameters of bacterial count in the yolk sac (yolk sac mesophiles (YSM) and yolk sac Enterobacteriaceae (YSE)) were evaluated based on six treatments with seven repetitions, and bacterial count on the eggshell (eggshell mesophiles (EGM) and eggshell Enterobacteriaceae (EGE)) were evaluated based on six treatments in triplicate.

The data were analyzed through multivariate analyses using SAS v.9.4 software (SAS Institute Inc., Cary, NC, USA). The analyses included correlations between incubation and microbiological variables, as well as principal component (PROC PRINCOMP), path, and cluster analyses. The paths (PROC CALIS) were defined based on the movement of effects of the internal and external factors affecting contamination and HI. Hierarchical clusters (PROC CLUSTER) were formed using the treatments, and then discrimination analyses (PROC STEPDISC and PROC DISCRIM) were used to define the factors that separated the clusters.

## 3. Results

The correlation analysis (Table 1) was conducted to elucidate the factors that influenced the HI rates. A significant negative correlation was identified between HI and EM (r = −0.79). Furthermore, it was found that EGM was strongly associated with YSM (r = 0.76) and YSE (r = 0.73), similar to EGE, which was also strongly correlated with YSM (r = 0.62) and YSE (r = 0.64). YSE was the main factor positively correlated with EM (r = 0.35), although this correlation was not statistically significant.

**Figure 1:**
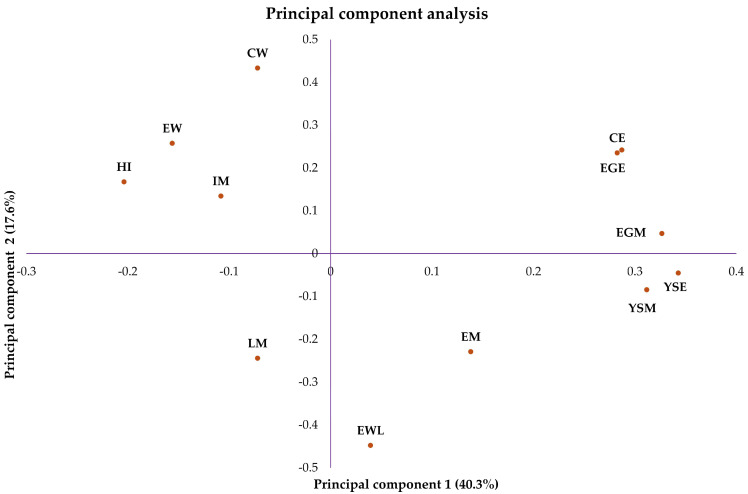
Principal component analysis of the parameters measured during the experimental period. Hatchability of fertile eggs (HI); number of contaminated eggs (CE); early mortality (EM); intermediate mortality (IM); late mortality (LM); egg weight (EW); egg weight loss (EWL); chick weight (CW); yolk sac mesophiles (YSM); yolk sac Enterobacteriaceae (YSE); eggshell mesophiles (EGM); eggshell Enterobacteriaceae (EGE).

In the principal component analysis, the first principal component accounted for 40.3% of the total variance, while the second principal component explained 17.6% of the variance (Figure 1). This structure revealed that HI was positioned opposite to variables associated with eggshell and yolk sac contamination, indicating a negative association between HI performance and contamination factors. Although this negative correlation was more pronounced between HI and the contamination variables, the spatial configuration also demonstrated that CW was oriented in the opposite direction to the contamination variables, suggesting a negative correlation. Although this remains a premature finding, it may suggest a possible indication that chicks with greater body weight tend to be associated with lower levels of contamination. CE exhibited a positive correlation with eggshell and yolk sac contamination, reinforcing that both sources of contamination are associated with CE during incubation.

**Figure 2:**
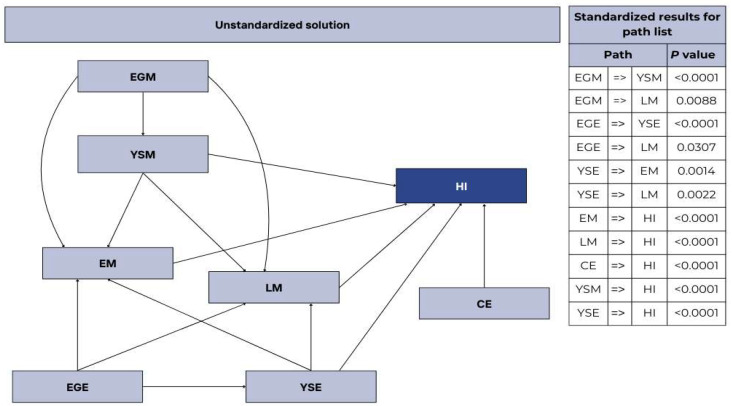
Path analysis of microbiological and incubation parameters with statistical significance. Hatchability of fertile eggs (HI); number of contaminated eggs (CE); early mortality (EM); late mortality (LM); yolk sac mesophiles (YSM); yolk sac Enterobacteriaceae (YSE); eggshell mesophiles (EGM); eggshell Enterobacteriaceae (EGE).

In the pathway diagram, we begin with the EGM, which represents the variable responsible for initiating the contamination effects in poultry (Figure 2). According to the proposed hypothetical model, contamination of the eggshell promoted contamination of the yolk sac, which in turn increased embryonic mortality and subsequently affected HI. The change in HI would, therefore, be a response to CE. In the diagram, we excluded pathways that were not statistically significant. This approach ensured a more accurate and clearer representation of significant relationships. The inclusion of statistically similar paths is not justified, as such relationships do not provide sufficient evidence to be considered relevant or discussed within the studied context. The results of the path analysis revealed that contamination of both the eggshell and yolk sac significantly influenced EM and LM. Additionally, the path analysis indicated that EM, LM, CE, YSM, and YSE significantly affected HI.

**Figure 3:**
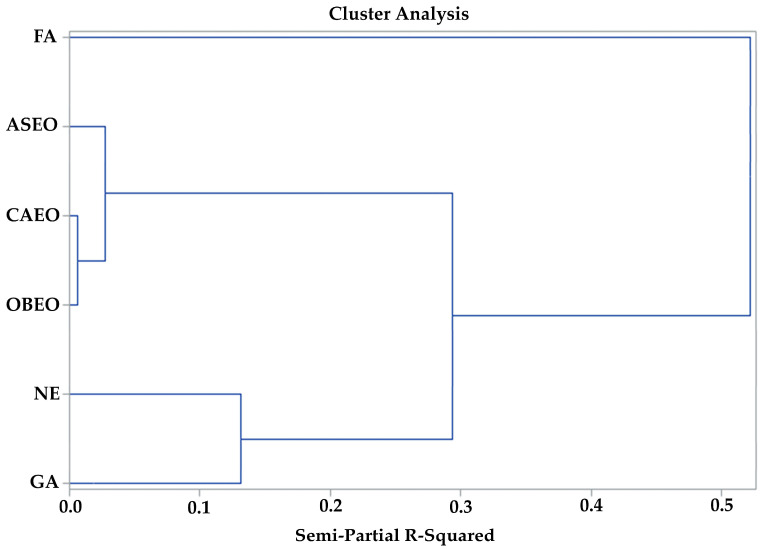
Hierarchical cluster analysis of the parameters measured during the experimental period. non-sanitized eggs (NE); grain alcohol (GA); formaldehyde (FA); *Citrus aurantifolia* essential oil (CAEO); *Ocimum basilicum* essential oil (OBEO); *Allium sativum* essential oil (ASEO).

The hierarchical cluster analysis, based on microbiological and incubation variables (Figure 3), classified the treatments into three distinct clusters. The treatment with FA formed an independent cluster (cluster 2), while the treatments with essential oils were grouped into a single cluster (cluster 3). The remaining treatments comprised Cluster 1. Discriminant analysis identified the variables responsible for separating the clusters, with EGE and EGM emerging as the primary determinants (Table 2). The strength of this separation is evidenced by the high partial R^2^ values, with EGE and EGM together accounting for more than 80% of the differentiation between cluster 1 and clusters 2 and 3. Notably, EGM alone contributed nearly 90% to the distinction between clusters 2 and 3. Furthermore, all analyzed variables were statistically significant in differentiating the clusters. These results suggest that the treatments promoted different decontamination and incubation performance profiles (Table 2).

In addition to the partial R^2^, which indicated the individual contribution of each variable to the separation of clusters, the values of *p* < *F*, *p* < Lambda, and *p* < ASCC were also analyzed (Table 2). The *p* < *F* showed the differences in means between groups, while *p* < Lambda and *p* < ASCC confirmed the multivariate separation of the clusters. All tests showed *p* < 0.05, indicating that the variables significantly discriminate the groups both individually and in combination.

**Figure 4:**
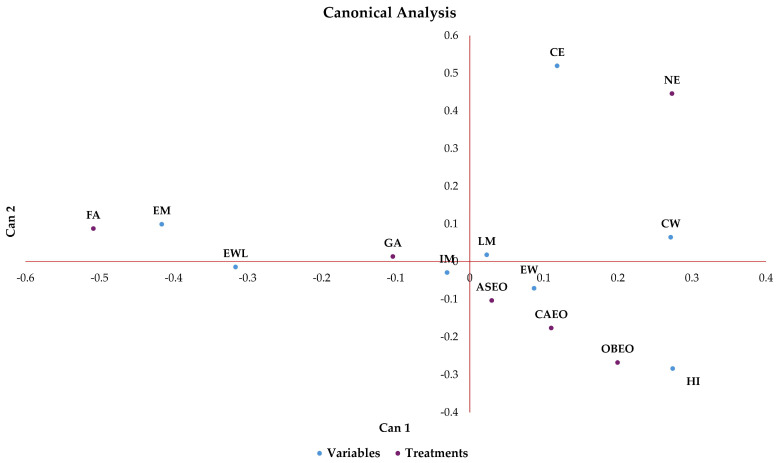
Canonical analysis of the parameters measured during the experimental period. Hatchability of fertile eggs (HI); number of contaminated eggs (CE); early mortality (EM); intermediate mortality (IM); late mortality (LM); egg weight (EW); egg weight loss (EWL); chick weight (CW); non-sanitized eggs (NE); grain alcohol (GA); formaldehyde (FA); *Citrus aurantifolia* essential oil (CAEO); *Ocimum basilicum* essential oil (OBEO); *Allium sativum* essential oil (ASEO).

Canonical correlation analysis revealed that the treatments with essential oils (CAEO, OBEO, and ASEO) were positioned close to HI and in the opposite direction of CE, indicating positive associations with HI and negative associations with CE (Figure 4). This configuration suggests that the application of essential oils promoted a reduction in CE and, consequently, an increase in HI. In contrast, the opposite positioning between the treatment with NE and the HI variable reveals a negative correlation, whereas the proximity between NE and CE, both located in the upper right quadrant of the graph, demonstrates a positive correlation. These results indicate that, unlike essential oils, the absence of sanitization results in a higher NE and a lower HI. The FA treatment, in turn, was positioned close to the EM suggesting a positive correlation between these factors, meaning that FA may be associated with increased EM. Furthermore, FA exhibited a negative correlation with both HI and CE, being oriented in the opposite direction to these variables. Although the reduction of CE with FA indicates sanitary efficiency, the lack of a positive association between FA and HI suggests that the bacterial control promoted by FA did not translate into improved HI. This result further supports the hypothesis that the positive correlation observed between FA and EM may have contributed to the decrease in HI, independently of the reduction in NE. This suggests that other contamination sources or factors related to the characteristics of the sanitizer may have played a role in the observed mortality.

The canonical structure revealed that, on the first axis (Can1), the separation of treatments was mainly explained by the variables EM (canonical coefficient = −0.416) and HI (canonical coefficient = 0.274) (Figure 4). On the second axis (Can2), CE (canonical coefficient = 0.519) exerted the greatest positive influence, while HI (canonical coefficient = −0.283) showed a negative influence.

**Figure 5:**
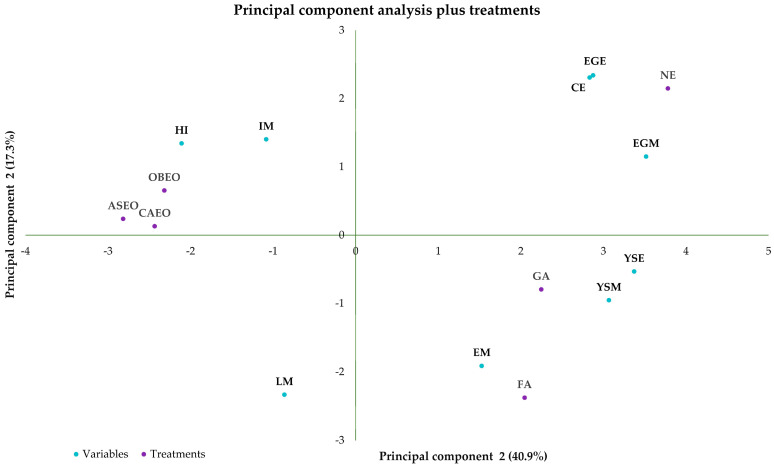
Principal component analysis of the parameters measured during the experimental period plus treatments. Hatchability of fertile eggs (HI); number of contaminated eggs (CE); early mortality (EM); intermediate mortality (IM); late mortality (LM); yolk sac mesophiles (YSM); yolk sac Enterobacteriaceae (YSE); eggshell mesophiles (EGM); eggshell Enterobacteriaceae (EGE); non-sanitized eggs (NE); grain alcohol (GA); formaldehyde (FA); *Citrus aurantifolia* essential oil (CAEO); *Ocimum basilicum* essential oil (OBEO); *Allium sativum* essential oil (ASEO).

Analyzing the principal component figure and treatments, it is observed that the first principal component accounted for 40.9% of the total variance, while the second principal component explained 17.3%. The treatments CAEO, OBEO, and ASEO exhibit a negative relationship with variables associated with contamination (CE, EGM, EGE, YSE, and YSM) (Figure 5). This distribution in the figure indicates that higher levels of these forms of contamination are associated with lower values in these treatments. In other words, ASEO, CAEO, and OBEO are linked to the reduction of the various forms of contamination analyzed, suggesting that these treatments may have a positive effect in decreasing the contaminant load and could be potentially effective within the studied sanitary control context. On the other hand, the FA treatment shows a strong positive correlation with the EM variable, as well as with YSM and YSE, both of which are related to yolk sac contamination and are positively correlated with EM. This indicates that the FA treatment is associated with higher levels of these contamination forms, which can be interpreted as an undesirable effect in the sanitary context. Additionally, FA presents a negative correlation with variables such as EC, EGM, and EGE, suggesting that in these aspects, the treatment may contribute to reducing contamination. Thus, FA tends to be less effective than essential oils in sanitary terms, as measured by YSM and YSE, and less efficient in productive terms, as measured by HI, since essential oils are positively correlated with HI and FA is negatively correlated with this variable.

## 4. Discussion

Our correlation analyses allow us to infer that contamination levels, both on the eggshell and in the yolk sac, directly or indirectly influence variation in HI. This variation due to eggshell contamination has been suggested by researchers from Brazil, Turkey, Egypt, the United States, and other countries [10,19,20,21] and can be explained by the dynamic between sanitized and non-sanitized hatching eggs, with non-sanitized hatching eggs often showing lower HI rates compared to those that are sanitized [15,22,23,24]. This occurs because embryos from non-sanitized hatching eggs have a significantly higher likelihood of exposure to bacterial contamination and subsequent infection [25]. Oliveira et al. [26] found a significant reduction of over 1.50 log_10_ in the bacterial load of the yolk sac of embryos from sanitized eggs, compared to embryos from non-sanitized eggs. Thus, the lack of proper egg sanitization, which maintains contamination and may promote an increase in CE during incubation, can represent an additional stress factor in poultry production.

The ASEO, CAEO, and OBEO treatments showed a negative correlation with problematic variables associated with contamination and reduced productivity (Figure 4 and Figure 5), indicating a greater potential to reduce embryonic mortality, mitigate the effects of contamination, and preserve HI. This finding can be supported by various studies, as evidenced in references [6,26,27,28,29,30,31,32]. Although eggshell contamination and CE showed a negative correlation with FA (Figure 4 and Figure 5), a positive correlation was observed with contamination-related variables associated with the yolk sac (Figure 5). This suggests a partial antibacterial effect, which may have impacted hatchability, given the observed negative correlation between FA and HI. Ogbu and Oguike [33] reported that reduced HI may be linked to several factors, including EM, egg rots, broken yolks, chicks dead-in-shell, prolonged storage before incubation, poor breeder nutrition, breeder age, and setters and hatchery malfunctions. Among these causes, we highlight EM, which was positively associated with FA sanitization in our study. Therefore, the negative correlation between FA and HI may be related to increased yolk sac contamination, which could have initially triggered embryonic mortality (Figure 4), in addition to the embryo toxicity of FA itself, which may lead to death during the early stages of development. The application of formaldehyde in setters should not be performed under any circumstances during the first four days of development, as it represents a risk to the embryos [34].

## 5. Conclusions

As global poultry production systems evolve to meet society’s demands increasingly safely and sustainably, identifying critical factors that threaten productivity and poultry health has become a priority. When identified early, these factors enable the implementation of proactive measures to prevent adverse impacts on systems managing hundreds of thousands or even millions of eggs and poultry daily. This study confirmed an inverse correlation between microbial load present on the eggshell and in the yolk sac, as well as CE during incubation and HI rates. In practical terms, the higher the level of contamination, the lower the HI. Notably, essential oils reaffirmed their potential as advanced sanitizing agents, proving effective in reducing both external and internal bacterial loads in hatching eggs. In contrast to formaldehyde, which was associated with increased EM and reduced HI, essential oils demonstrated beneficial effects, positioning them as a safer and more efficient solution for microbial control. This research raises a warning flag regarding the toxicity of FA to embryonic survival. Ultimately, the use of essential oil-based sanitizers is recommended for efficiently sanitizing hatching eggs in poultry production.

## Figures and Tables

**Table 1:** Correlation between the analyzed variables.

	CE	EM	IM	LM	EW	EWL	CW	YSM	YSE	EGM	EGE
HI	−0.35 ns	−0.79 ****	−0.10 ns	−0.25 ns	0.03 ns	−0.18 ns	0.08 ns	−0.30 ns	−0.47 ns	−0.37 ns	−0.17 ns
CE		0.11 ns	−0.08 ns	−0.31 ns	−0.03 ns	−0.25 ns	0.30 ns	0.49 *	0.71 ***	0.70 **	0.61 **
EM			−0.04 ns	−0.19 ns	−0.15 ns	0.24 ns	−0.35 ns	0.18 ns	0.35 ns	0.04 ns	0.04 ns
IM				−0.12 ns	0.15 ns	0.09 ns	0.23 ns	−0.23 ns	−0.22 ns	−0.17 ns	−0.11 ns
LM					0.14 ns	0.11 ns	0.06 ns	−0.11 ns	−0.31 ns	0.03 ns	−0.29 ns
EW						0.18 ns	0.63 ***	−0.48 *	−0.42 ns	−0.35 ns	−0.38 ns
EWL							−0.37 ns	0.30 ns	0.20 ns	0.05 ns	−0.35 ns
CW								−0.37 ns	−0.16 ns	−0.13 ns	0.08 ns
YSM									0.82 ****	0.76 ***	0.62 **
YSE										0.73 ***	0.64 **
EGM											0.79 ****

Abbreviations: hatchability of fertile eggs (HI); number of contaminated eggs (CE); early mortality (EM); intermediate mortality (IM); late mortality (LM); egg weight (EW); egg weight loss (EWL); chick weight (CW); yolk sac mesophiles (YSM); yolk sac Enterobacteriaceae (YSE); eggshell mesophiles (EGM); eggshell Enterobacteriaceae (EGE), not significant (ns); * *p* < 0.05, ** *p* < 0.01, *** *p* < 0.001, **** *p* < 0.0001.

**Table 2:** Discriminant analysis of the variables responsible for separating the clusters.

Cluster 1 vs. Clusters 2 and 3				
Variables	R^2^ Partial	*p* < *F*	*p* < Lambda	*p* < ASCC
EGE	0.8086	0.0011	<0.0001	<0.0001
EGM	0.8068	0.0024	<0.0001	0.0017
CE	0.7918	0.0067	<0.0001	<0.0001
EM	0.7694	0.0188	<0.0001	0.0002
EWL	0.7513	0.0432	<0.0001	<0.0001
Cluster 2 vs. cluster 3				
EGM	0.8813	0.0005	0.0005	0.0005

Abbreviations: Eggshell Enterobacteriaceae (EGE); eggshell mesophiles (EGM); number of contaminated eggs (CE); early mortality (EM); egg weight loss (EWL). Coefficient of determination (R^2^); Tests for multivariate separation of clusters (*p* < Lambda and *p* < ASCC).

## Data Availability

The raw data supporting the conclusions of this article will be made available by the authors on request.
